# The Study of Humic Substances’ Impact on Anion Exchangers

**DOI:** 10.3390/ma17061237

**Published:** 2024-03-07

**Authors:** Paweł Wiercik, Tomasz Garbowski, Przemysław Chrobot

**Affiliations:** 1 The Faculty of Environmental Engineering and Geodesy, Institute of Environmental Engineering, Wrocław University of Environmental and Life Sciences, Grunwaldzki Square 24, 50-363 Wrocław, Poland; 2 Institute of Technology and Life Sciences–National Research Institute Falenty, 3 Hrabska Avenue, 05-090 Raszyn, Poland; t.garbowski@itp.edu.pl; 3 Municipal Water and Sewage Company Wrocław, 14/16 Na Grobli St., 50-421 Wrocław, Poland; przemyslaw.chrobot@mpwik.wroc.pl

**Keywords:** humic substances, infrared spectroscopy, anion exchanger, organic fouling

## Abstract

Humic substances (HSs) present in water and wastewater cause fouling of anion exchange resins (AERs), which mainly results in reducing the ion exchange capacity (IEC). In this paper, an attempt was made to investigate fouling of two polystyrene and one polyacrylic AER using water from the Oder River, treated wastewater after the ultrafiltration process (UFTW) and digester reject water from sludge dewatering at the Janówek Wastewater Treatment Plant (WWTP) in Wrocław. HSs contained in digester reject water were characterised by the lowest aromaticity and molecular weights (MWs), the highest proportion of hydrophilic fraction and the highest amount of oxygenated functional groups. The Fourier-transform infrared (FTIR) analyses made it possible to identify chemical bonds characteristic of HSs and determine the mechanism of their retention on the surface of AER beads. The conducted experiments brought unexpected results, as the IEC increased with the amount of organic matter in the feed. Presumably, the humic substances accumulated on the beads and in the porosity of the anion exchangers themselves participated in the ion exchange process.

## 1. Introduction

The presence of HSs in water sources and wastewater influences treatment processes. HSs are polymers formed by microbial decomposition of plant and animal residues. Their structures can be described as assemblies of covalently linked aromatic and aliphatic residues carrying mainly carboxyl, phenolic and alkoxy groups [1,2,3], which cause their overall charge to be negative. HSs, together with low MW acids, proteins, amino acids and carbohydrates, constitute the natural organic matter (NOM) in all drinking water sources [4]. One of the methods applied to remove NOM is IE [5]. It has been established that IE preferentially removes NOM of small and medium molecular size, which is difficult to remove by coagulation [6,7,8].

One of the factors that has a significant effect on the adsorption of organics is the resin matrix. Polystyrene AERs have a strong affinity for anionic organics with hydrophobic groups because of their hydrophobic interaction, whereas polyacrylic AERs exhibit a greater capacity for NOM. Polyacrylic resins tend to have a more open structure and higher water content. They are more resistant to fouling with higher regeneration efficiency because of their hydrophilic structure [9,10]. The pore structure of AERs is also important for NOM removal. Gel-containing resins have no permanent pores, but they do have micropores of atomic size. Macroporous resins, on the other hand, have a true pore phase in addition to the gel phase, and are therefore more accessible to organics of large molecular sizes than gel-containing resins [11]. However, research by J.-P. Croue et al. [7] showed that a greater degree of dissolved organic carbon (DOC) removal occurred on the AER with a gelular structure than on the macroporous one. As stated by P. L. K. Fu and J. M. Symons [11], the resin skeleton plays a more important role than the pore structure in terms of organics removal by IE. Therefore, the polyacrylic resins removed more aquatic organics than polystyrene ones (even macroporous). Due to the fact that HSs exhibit both hydrophilic and hydrophobic properties, the selection, optimisation and preparation of AERs for their removal is difficult.

The main problems concerning water treatment plants which remove NOM via anion exchange include pretreatment requirements, loss of capacity over time and more frequent regeneration than expected [6]. The capacity loss of AERs results from their susceptibility to organic fouling. The negatively charged ions of the aliphatic side chains of HSs are exchanged for mobile anions (e.g., chloride or hydroxyl ions) connected with the functional groups of AERs. The rest of their structure accumulates on the surface of the AER bead, inhibiting the penetration of counterions into the resin and interrupting the exchange sites [2,12,13,14,15]. The hydrophobic moieties of the organic molecule may also adsorb onto the resin polymer. Consequently, the AER becomes hydrophobic and its moisture, porosity and IEC decrease [16]. The organic molecule can be removed either by the IE mechanism alone, by the surface adsorption mechanism alone, or by a combination of both mechanisms. When both mechanisms are involved, the anionic groups of the organic molecule bind with the resin amine functional groups (IE mechanism) and the nonionic part of the molecule attaches to the inner surface of the resin by surface adsorption [11].

In general, the performance of the AER is assessed based on the breakthrough curves of different anions and other water quality parameters (e.g., pH, conductivity, turbidity, DOC, etc.) [17]. The pH titration curve and the study of adsorption kinetics are other means of characterising an ion exchanger [18]. The organic fouling of the AERs can be studied using the instrumental methods which enable to detect or to quantitatively determine the foulants. The most commonly applied instrument to measure the content of organic carbon species is the total organic carbon (TOC) analyser. UV and UV-vis spectrophotometers provide information on the UV254 and UV absorbance. Fluorescence spectrophotometers and chromatographs provide information on dissolved organic carbon [2,15]. Another method which allows the identification of carbon atoms in organic molecule is ^13^C-NMR (carbon-13 nuclear magnetic resonance) spectroscopy [19].

To the authors’ knowledge, there is little literature on the application of FTIR spectroscopy to study the changes in the resin beads surface during its exploitation. FTIR has been used to evaluate the performance of the resins during the softening process [20], to determine the functional groups in the resin structure responsible for fluoride sorption [21] and to determine the thermal degradation steps of the analysed resins [22]. As the author’s previous research has shown [2,15], FTIR is quick and cost-effective method for detecting foulants and observing changes on the surface of AER beads during IE.

In this study, an attempt was made to investigate the influence of HSs contained in the Oder River water, UFTW and digester reject water from dewatered sewage sludge on the fouling of selected AERs. For this purpose, analyses of selected physicochemical water quality parameters (UV254, chemical oxygen demand (COD), TOC, chlorides, turbidity, colour, dry residue, pH) in samples of raw medium and filtrates were performed. The fresh, exhausted and regenerated resin beads from each IE cycle were also examined using the Nicolet iN10 integrated infrared microscope with Nicolet iZ10 external FTIR module. The IEC was determined on the basis of the difference in chloride content between the regenerant used (10% NaCl) and the eluate. This is a new approach to the problem, as in practice the loss of the IEC in AERs is controlled by analysing the change in the amount of anions removed per one unit of AER in subsequent service cycles.

In the literature, there is a paucity of research concerning organic fouling of AERs conducted on real wastewater. On the contrary, there are numerous laboratory tests conducted on synthetically prepared solutions or organic matter isolates [6,9,23]. Taking this into account, it is difficult to predict how other compounds and anions present in the real water or wastewater would affect the fouling of AERs. To the authors’ knowledge, using FTIR spectroscopy to assess organic fouling of AERs is also a novel approach to this problem.

## 2. Materials and Methods

The research on the effect of HSs on the fouling of AERs was conducted using Lewatit S 5428 (macroporous, polyacrylic), Lewatit S 6328 A (macroporous, polystyrene) and Lewatit MonoPlus M 500 (gelular, polystyrene) AERs produced by Lanxess Deutschland GmbH (Leverkusen, Germany), with a total IEC of min. 0.85 eq/L, min. 1 eq/L and min. 1.2 eq/L (based on resin safety data sheets), respectively. The functional groups of these AERs are quaternary amines (type 1).

Water from the Oder River, UFTW and digester reject water from sludge dewatering on the filter presses at the Janówek WWTP in Wrocław (Poland) were used in these experiments. In total, 8 IE cycles on UFTW, 8 IE cycles on the Oder River water and 5 IE cycles on digester reject water were carried out on Lewatit S 5428. 8 IE cycles on UFTW and 8 IE cycles on the Oder River water were carried out on Lewatit S 6328 A. A total of 8 IE cycles on UFTW and 5 IE cycles on digester reject water were carried out on Lewatit MonoPlus M 500. 

Each medium was collected in the IE feed (100 L, steel), from which it was fed through the IE column with the given resin using the Watson-Marlow Qdos 30 Profibus peristaltic pump. The research was conducted on the IE system at Janówek WWTP Test Station in Wrocław (Wrocław, Poland). The IE service cycles were carried out until 40 bed volumes (BV) of the medium had passed through the column. The exception was the filtrate samples from the first IE cycle, which was carried out until 60 BV of the medium had passed through the column. In the experiment with Lewatit S 5428, when the feed was water from the Oder River, the first cycle was carried out until 75 BV. Samples of the filtrate from the column were taken for physicochemical analysis every 4 BV, except for the first IE, when samples were taken every 8 BV. In the Oder River water experiment with Lewatit S 5428, the filtrates were collected every 5 BV and every 10 BV in the first cycle. Co-current regeneration was then performed using 10% NaCl. The resin bed was first rinsed with distilled water, then 10% NaCl was introduced at a flow rate of 5 BV/h, and finally the residual brine was flushed out with distilled water. Samples of the eluate were collected during the sodium chloride rinsing.

Physicochemical analyses of the selected water quality parameters (UV_254_, COD, TOC, chlorides, turbidity, colour, dry residue, pH) in the samples of raw medium and filtrates were performed. The IEC was determined on the basis of the difference in chloride content between the regenerant used (10% NaCl) and the eluate and was calculated from Equation (1):

(1) $$IEC=\frac{Q\cdot C}{BV}$$

where *IEC*—ion exchange capacity [eq/L]; *Q*—amount of regenerant used [m^3^]; *C*—chloride concentration as the difference between the amount of chloride introduced with 10% NaCl and the amount of chloride in the eluate [eq/m^3^]; *BV*—bed volume [L].

In order to know the properties of the HSs contained in the tested media, the quotient E_2_/E_3_ (absorbances at 250 nm and 365 nm) and aromaticity were calculated. The relationship between aromaticity and this quotient for the isolated humic fractions according to J. Peuravuori and K. Pihlaja [24] is:

aromaticity [%] = 52.509 − 6.78 E_2_/E_3_, (2)

The chloride concentration was determined by Mohr’s method. The regenerant and eluate samples were properly diluted before titration with silver nitrate to avoid the interference from other ions or compounds.

TOC measurement was performed using a Sievers InnovOx Laboratory TOC Analyzer with GE Autosampler (GE Analytical Instruments, Boulder, CO, USA) and UV_254_ was measured using a Thermo Scientific UV-Vis spectrophotometer Evolution 201 (Thermo Fisher Scientific Inc., Madison, WI, USA). The fresh, exhausted and regenerated resin beads were also analysed by means of FTIR spectroscopy. Attenuated total reflection-Fourier transform infrared (ATR-FTIR) analyses were performed using the Nicolet iN10 integrated infrared microscope with the Nicolet iZ10 external FT-IR module (Thermo Fisher Scientific Inc., Madison, WI, USA). Each spectrum was the average of 32 scans in the 400–4000 cm^−1^ wavenumber range at the 4 cm^−1^ spectral resolution.

## 3. Results

The physicochemical composition of the Oder River water, UFTW and digester reject water used to foul Lewatit S 5428, Lewatit S 6328 A and Lewatit Monoplus M 500 resins is presented in Table 1.

It can be seen that during the storage of a given medium in the tank, chemical reactions and partial sedimentation of suspensions occurred. At the moment of starting the pump before the following IE cycle, the suspensions were to some extent moved from the bottom of the tank, which can explain the significant differences in the composition of the treated medium. Of the analysed samples, the highest content of HSs was found in the digester reject water samples, as evidenced primarily by the colour, COD, dry residue (organic) and UV_254_ absorbance.

The colour of Lewatit S 5428 and Lewatit S 6328 A beads changed during the IE process and partly remained in this form after regeneration. In the case of Lewatit MonoPlus M 500, the colour of the beads changed to dark brown (UFTW experiment) and black (digester reject water experiment) and remained after regeneration from the first IE cycle, indicating organic fouling, which was irreversible.

**Figure 1:**
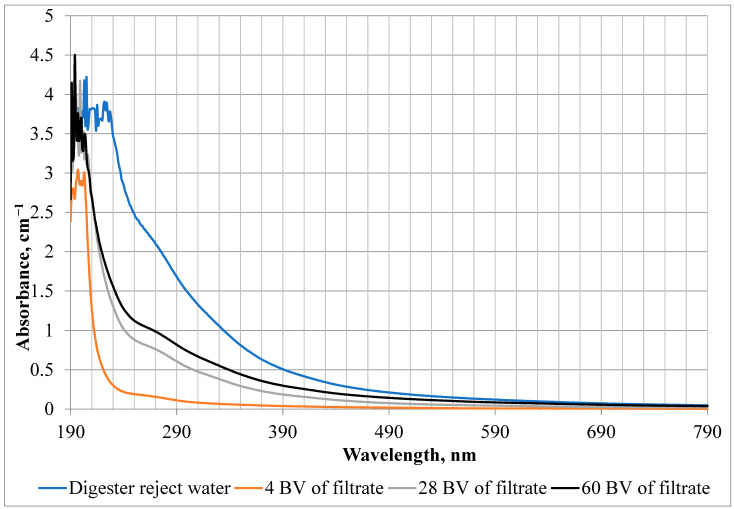
UV-Vis spectra of digester reject water and filtrate samples from the 1st cycle of IE with Lewatit S 5428.

An analysis of the UV-Vis spectra was also performed, an example of which is shown in Figure 1. The shape of the river water and UFTW spectra, as well as the UV absorbance values were similar in the experiments conducted with Lewatit S 5428 and Lewatit S 6328 A. All spectra increase almost uniformly in intensity from 770 to 210 nm. The spectra of digester reject water are very similar to the spectra of humic acids, which have a discreet “shoulder” observed at around 280–270 nm [25]. This is the result of overlapping absorbance of a large number of chromophores present in the humic core. In general, the chromophores responsible for the absorption in the UV region are principally phenolic arenes, benzoic acids, aniline derivatives, polyenes, and polycyclic aromatic hydrocarbons with two or more rings [25].

It can be seen that HSs were present in the first IE cycle filtrate samples on Lewatit S 5428 resin, but were also retained on its beads, as evidenced by lower UV absorbance values compared to the digester reject water sample. A similar relationship in the case of filtrates was observed for the Oder River water and the UFTW passed through the selected AERs. The spectra of the Oder River water and the UFTW are similar in shape to the spectra of humic and fulvic acids obtained by H. Niu et al. [26].

**Figure 2:**
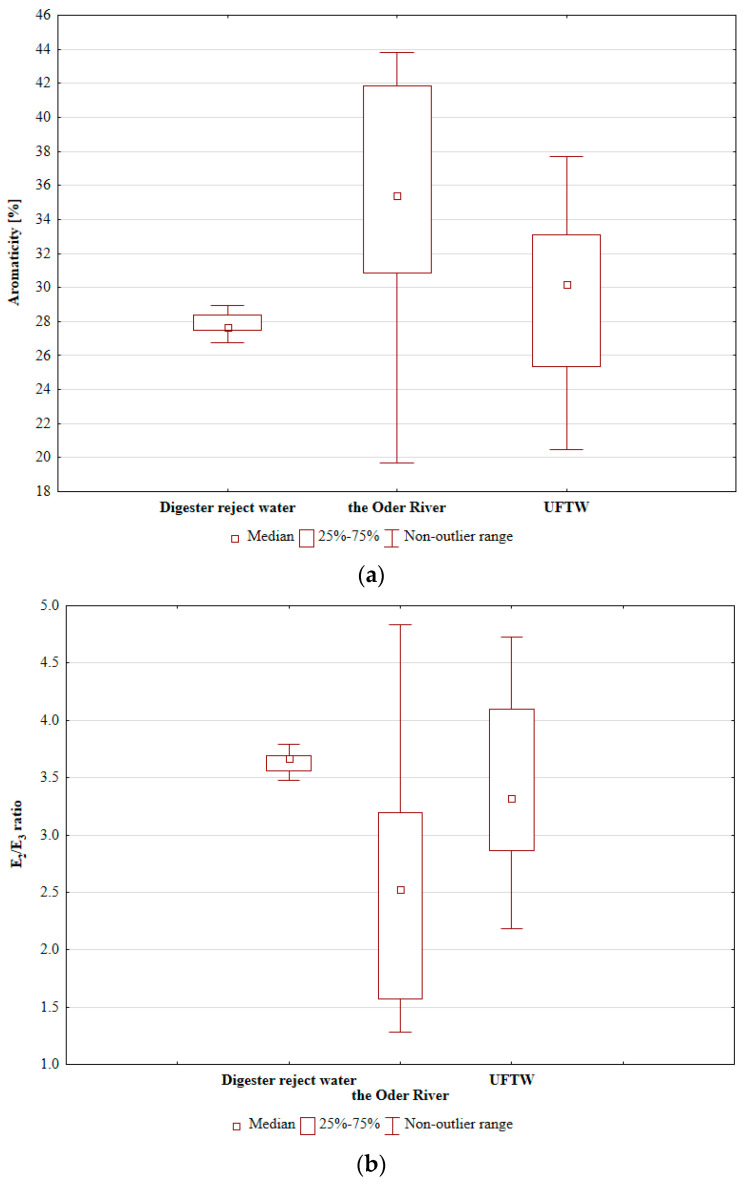
Percentage aromaticity (**a**) and E2/E3 ratio (**b**) of HSs present in the given media treated in subsequent IE cycles.

Figure 2 shows the aromaticity and the E_2_/E_3_ quotient of HSs in treated media. As the E_2_/E_3_ quotient increases, the aromaticity and molecular size of aquatic humic solutes decrease [24].

**Figure 3:**
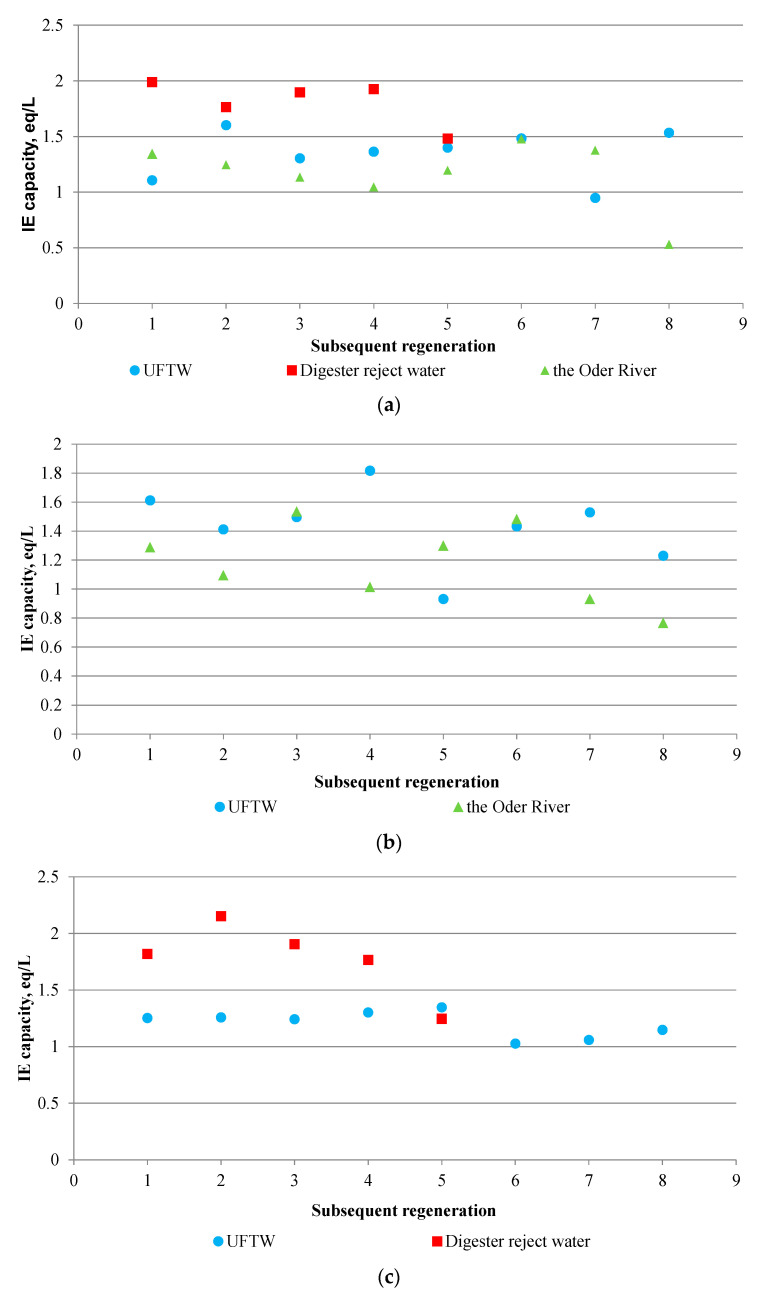
Changes in IEC in subsequent cycles in research conducted on (**a**), Lewatit S 6328 A resin, (**b**) Lewatit S 5428 resin, (**c**) Lewatit MonoPlus M 500 resin.

J.-P. Croue et al. [7] determined that the higher the hydrophilic character of the NOM fraction, the higher the COOH content, the lower the aromatic carbon content and the lower the MW. Analysing the data presented in Figure 2, it can be concluded that HSs in digester reject water had the lowest aromaticity, thus their MW was lower and they were more hydrophilic with a higher content of COOH functional groups compared to HSs in river water or UFTW. Figure 3 shows how the IEC changed during the research on the given AERs.

Before starting the research, it was assumed that the presence of HSs would have a negative effect on the IE process, i.e., that as the amount of these substances in the treated medium increased, the IEC of the AER would decrease. This is a result of the fact that the negatively charged ions of the aliphatic side chains of HSs are exchanged for mobile anions connected with the functional groups of AERs. The rest of their structure accumulates on the surface of the AER bead, inhibiting the penetration of counterions into the resin and interrupting the exchange sites. Therefore, a larger amount of HSs in the treated medium should result in greater fouling of AER and thus lower IEC values in subsequent IE cycles. However, the conducted research brought unexpected results. It appeared that despite the fact that HSs accumulated on the AER beads and reduced the penetration of ions from the treated medium into their interior, this did not result in the determination of low IEC values. The IEC in individual tests for almost all IE cycles oscillated around higher values than those provided by the manufacturer. In addition, the IEC was very variable from one cycle to another, making it impossible to determine the trend of these changes. It can therefore be concluded that it was not possible, on the basis of the IEC values obtained, to indicate the point at which the use of a given AER would be uneconomical.

Much higher values of IEC were found in the experiments with digester reject water than in the experiments with river water or UFTW. In the experiments with digester reject water, the IEC in each cycle was higher than specified by the manufacturer. Taking into account the fact that in other experiments there were cycles where the IEC was lower than specified by the manufacturer, it can be assumed that HSs in the first phase of IE (several cycles) did not interfere with process.

However, in the long term (several dozen cycles), it is possible that their negative impact on the IE process would be marked. The impact of these substances turned out to be more complex than expected, therefore the IE process should be carried out for longer—not for several, but for several dozen cycles. Most likely, the HSs accumulated on the beads and in the porosity of the AERs themselves participated in the IE process, because the fouled AER beads either did not participate in the IE process or their participation was negligible, which resulted from the fact that the exchange sites were largely occupied by negative ions of the HSs structures. This would indicate that the negatively charged ions contained in the HSs chains accumulated on the surface of the AER beads were exchanged for ions from the treated medium. During the regeneration with the sodium chloride solution, the chlorides penetrated not only into the anionite beads, but also into the structures of the HS, causing a lower concentration of chlorides in the eluates. Therefore, the calculation of the IEC on the basis of the difference between the amount of chlorides introduced into the ion exchanger during regeneration and the amount of chlorides in the eluate did not really give information about the amount of chlorides that were introduced only to the AER beads, but also about those that entered the structure of HS.

This thesis is also supported by the analysis of the chloride content in the filtrates during the IE process. When river water or UFTW passed through the column, more-or-less similar amounts of chlorides were detected in the filtrates from the very beginning of a given IE cycle, at a level about 2–3 times higher than in the raw medium. In these experiments, where the feed was digester reject water, chloride concentrations of more than 2000 mg/L were detected in the filtrates from the beginning of each IE cycle. High chloride concentrations of about 1000–1500 mg/L were obtained in the filtrates up to about 20 BV.

In the case of tests carried out on digester reject water, which contained a higher amount of HSs with hydrophilic fraction and oxygenated functional groups than HSs in river water and UFTW, and the reaction in the filtrates oscillated around pH 9.0, it can be concluded that the dominant mechanism was IE. Due to the higher amount of oxygenated functional groups in the HSs structure, a higher amount of chlorides was exchanged in the IE. This is confirmed by the research of J.-P. Croue et al. [7], who showed that with increasing pH, more chlorides were exchanged and the NOM structures became more negatively charged, which favoured IE.

More chlorides were exchanged in the case of the hydrophilic fraction with an alkaline reaction, which is related to its higher content of oxygenated functional groups. According to J.-P. Croue et al. [7], in an acidic environment, the retention of the hydrophobic fraction on the resin did not lead to the release of chlorides, suggesting that adsorption was the dominant mechanism, whereas in a neutral reaction both mechanisms took place. This also explains the lower amount of chlorides in the filtrates in the river water and UFTW tests, where the reaction was lower-in the filtrates it oscillated around slightly acidic and neutral values.

**Figure 4:**
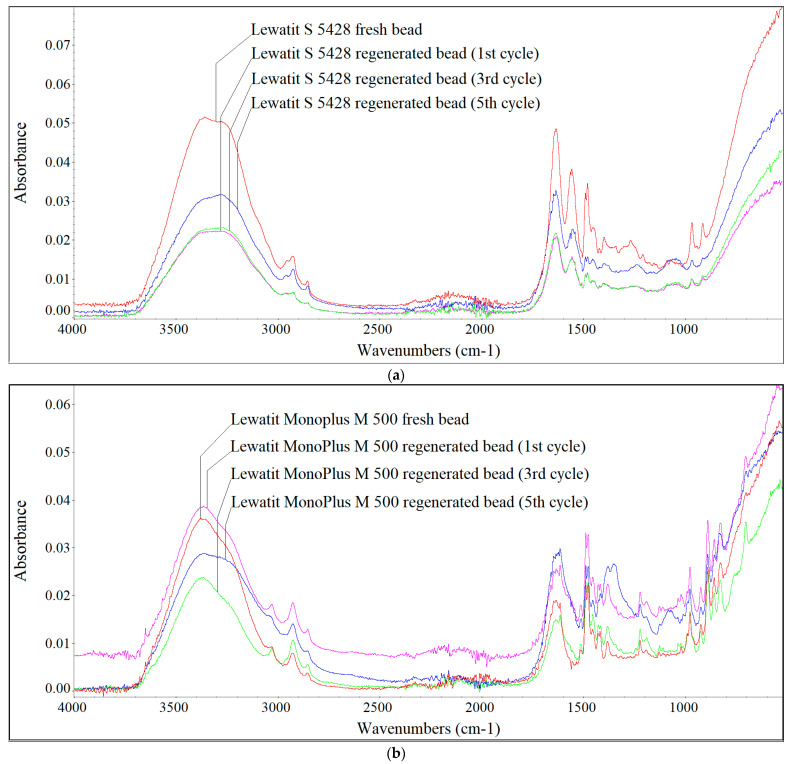
FTIR spectra of the fresh and regenerated (**a**) Lewatit S 5428 and (**b**) Lewatit MonoPlus M 500 beads fed with digester reject water.

**Figure 5:**
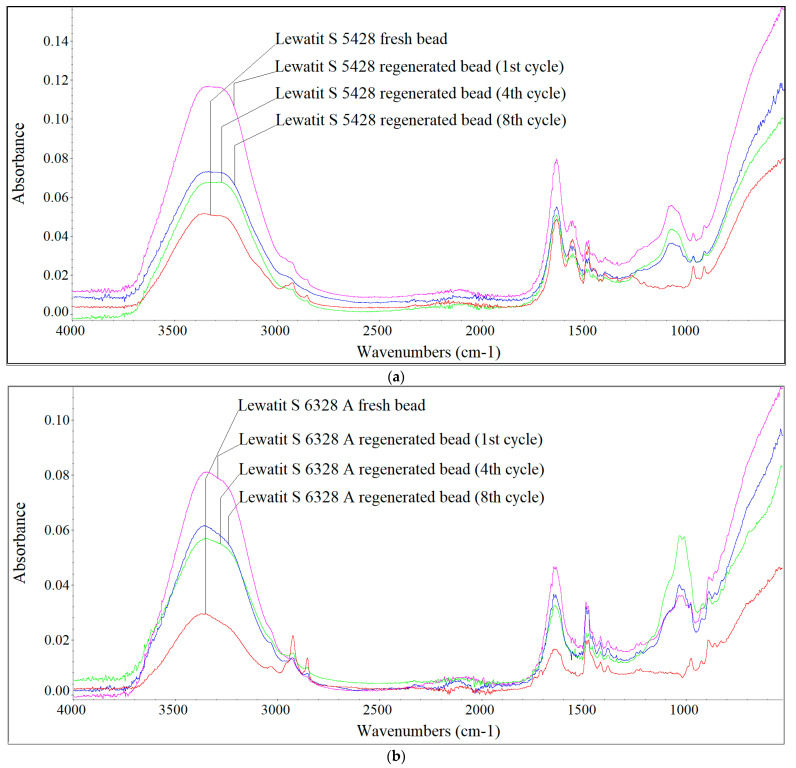
FTIR spectra of the fresh and regenerated (**a**) Lewatit S 5428 and (**b**) Lewatit S 6328 A beads fed with the Oder River water.

**Figure 6:**
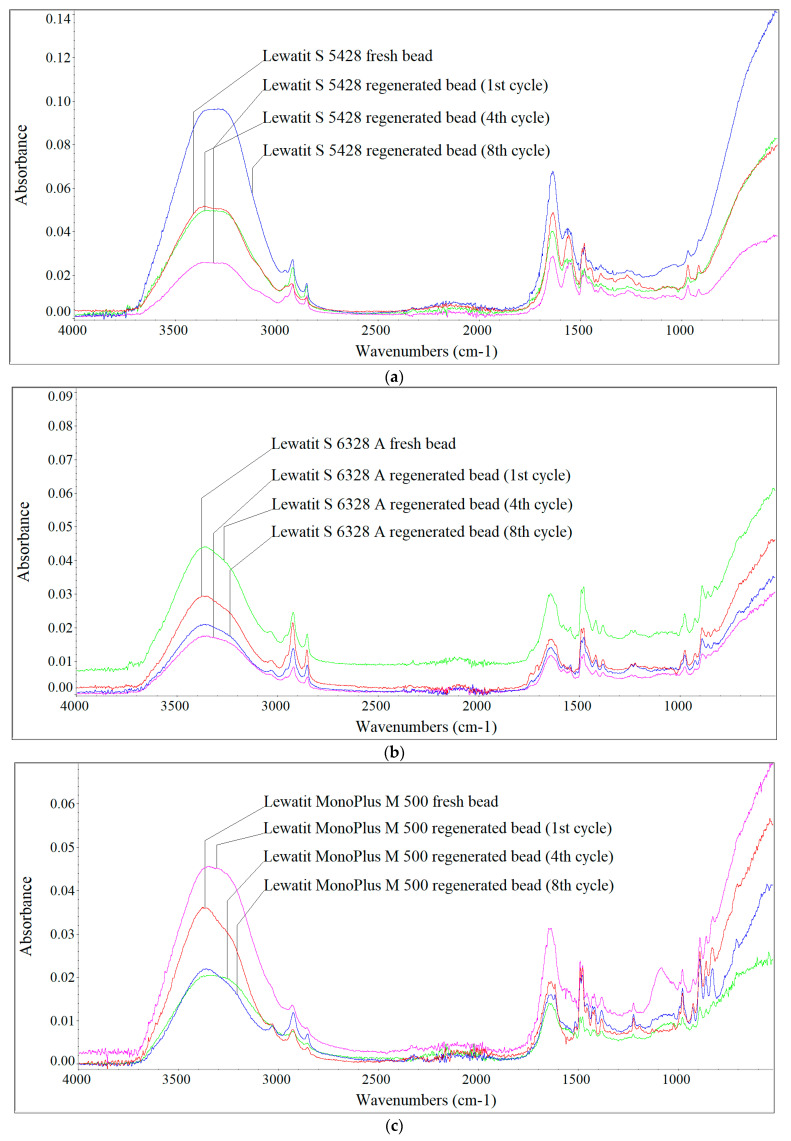
FTIR spectra of the fresh and regenerated (**a**) Lewatit S 5428, (**b**) Lewatit S 6328 A, (**c**) Lewatit MonoPlus M 500 beads fed with the UFTW.

Figure 4, Figure 5 and Figure 6 show the FTIR spectra of fresh and regenerated beads of individual AERs fed with digester reject water, the Oder River water and UFTW.

Analysing the qualitative changes on the surface of the AER beads, it can be seen that the largest changes were caused by the HSs contained in the river water, which were characterised by the largest proportion of the hydrophobic fraction with the highest MW. In the case of HSs contained in digester reject water, due to their smaller sizes (lower MWs) and greater hydrophilicity, they were mainly retained in the porosity of the Lewatit S 5428 resin, therefore, slight changes in the shape of the spectra for fresh and regenerated beads were observed. In the case of the gelular Lewatit MonoPlus M 500 resin, the accumulation of HSs from digester reject water on the surface of the beads (Figure 4) was also detected, which was mainly evidenced by peaks at wavenumbers 1378 cm^−1^, 1081 cm^−1^, 1070 cm^−1^ and 1036 cm^−1^ (values not shown in the figures). According to Z.-H. Qin et al. [27] and X. Chen et al. [28] the C–H bending and symmetric CH_3_ deformation appear at 1380 cm^−1^. The peaks at 1081 cm^−1^, 1070 cm^−1^ and 1036 cm^−1^ are assigned to C–O in alcohols and aliphatic ethers [29]. According to [30] the peak at 1039 cm^−1^ can also be attributed to O–H stretching in polysaccharides.

Sodium chloride did not remove these compounds, which was confirmed by the increased chloride uptake during regeneration. This falsified the results of the IEC calculated on the basis of chloride content. The HSs present in the river water, characterised by higher MW and greater hydrophobicity, caused the accumulation of these fractions on the AER beads (Figure 5), which was primarily manifested by differences in absorbance intensity at wavenumbers around 1540 cm^−1^, 1235 cm^−1^, approximately 1160 cm^−1^, 1080 cm^−1^, 1065 cm^−1^, 1026 cm^−1^ and 1001 cm^−1^ (values not shown in the figures). The absorption band at 1540 cm^−1^ indicates N–H structures [29], 1235 cm^−1^ C–O stretching and O–H bending of COOH groups and phenols [30], 1160 cm^−1^ C–O stretching of aryl esters [30]. In the case of the research conducted on the Oder River water, the greatest changes in the shape of the spectra were observed in the wavenumber range of 1100–1000 cm^−1^, at which, as previously mentioned, the C–O groups in alcohols and aliphatic ethers and the O–H stretching in polysaccharides are identified. UFTW was characterised by a lower HSs aromaticity than the Oder River water, and at a lower pH, besides IE, adsorption of hydrophobic fractions also occurred on polystyrene AERs Lewatit S 6328 A and Lewatit MonoPlus M 500 (Figure 6). The greatest changes in the shape of the spectra were observed at the wavenumbers around 1555 cm^−1^ (the presence of N–H groups), 1081 cm^−1^ and 1011 cm^−1^ (the presence of C–O groups in alcohols and aliphatic ethers and the O–H stretching in polysaccharides). The surface of polyacrylic Lewatit S 5428 resin beads was the least susceptible to HSs accumulation contained in UFTW, therefore slight differences in the shape of the spectra were observed (Figure 6c).

## 4. Conclusions

The type of AER (its matrix, pore structure), the quantity and quality of organic compounds in the treated medium, as well as the conditions in which the IE process takes place determine the mechanism of HSs retention by the AER. This causes the organic fouling phenomenon to be complex. As this and authors’ previous research have shown, FTIR is a quick and effective method not only for identification, but also for assessing the ageing process, which is a significant achievement in this field, especially since it is not commonly used for this purpose. In the current research, physicochemical analyses and UV-Vis spectroscopy were additionally performed. The UV-Vis spectra of the treated media and filtrates were similar in shape to the spectra of humic and fulvic acids. These studies made it possible to learn the characteristics of the HSs present in the treated media. Compared to the HSs in the Oder River water and UFTW, the HSs present in digester reject water were characterised by the lowest aromaticity, and thus the lowest MW, the highest proportion of the hydrophilic fraction, and the largest amount of oxygenated functional groups (COOH). The dominant mechanism of HSs retention in the experiments on digester reject water was IE. The negatively charged ions of the aliphatic side chains of HSs were exchanged for mobile anions connected with the functional groups of AERs. The rest of their structure accumulated on the surface of the AER beads. The HSs were largely retained in the porosity of the macroporous Lewatit S 5428 resin, and in the case of the gelular Lewatit MonoPlus M 500 resin also on the surface of its beads. Greater hydrophobicity, aromaticity and MW of HSs contained in the Oder River water and UFTW meant that the main mechanism of their retention was both IE and adsorption.

The performed research sheds new light on the behaviour of HSs in the IE process. It is not excluded that HSs may take part in IE, as evidenced by the surprisingly high values of IEC of heavily fouled AERs after regeneration with sodium chloride. It can be assumed that chlorides during regeneration penetrated not only into the AER beads, but also into the HS structures accumulated on them, thus overestimating the calculated IEC. However, the issue of the participation of HSs in the IE process requires verification and further research.

## Figures and Tables

**Table 1:** Chemical constitution of media treated with Lewatit S 5428, Lewatit S 6328 A and Lewatit Monoplus M 500 resins.

Parameter	the Oder River	UFTW	Digester Reject Water
pH (-)	7.8–8.5	7.6–8.8	8.6–8.9
Turbidity (NTU)	0.43–44.2	0.34–9.4	58.7–90
Colour (mg Pt/L)	10–30	25–40	100–250 *
COD (mg O_2_/L)	31.8–157.8	13.2–121.1	273.1–568.2
Chloride (mg Cl^−^/L)	122–376	155.6–296	269–540
Dry residue (general) (mg/L)	415–865	650–1140	1275–1360
Dry residue (mineral) (mg/L)	160–585	248–555	395–505
Dry residue (organic) (mg/L)	245–370	295–600	790–905
UV_254_ absorbance (cm^−1^)	0.112–0.675	0.146–0.331	2.365–2.758
TOC (ppm)	8.09–39.1	8.42–13.8	not measured

* colour exceeded the highest value of 250 mg Pt/L.

## Data Availability

Data are contained within the article.
